# Engagement in a pilot produce prescription program in rural and urban counties in the Southeast United States

**DOI:** 10.3389/fpubh.2024.1390737

**Published:** 2024-06-10

**Authors:** Caroline E. Owens, Miranda Cook, Tammy Reasoner, Aleta McLean, Amy Webb Girard

**Affiliations:** ^1^Department of Anthropology, Washington State University, Pullman, WA, United States; ^2^Department of Anthropology, Emory University, Atlanta, GA, United States; ^3^Laney Graduate School, Emory University, Atlanta, GA, United States; ^4^Open Hand Atlanta, Atlanta, GA, United States; ^5^Urban Health Institute, Emory University, Atlanta, GA, United States; ^6^Hubert Department of Global Health, Rollins School of Public Health, Emory University, Atlanta, GA, United States

**Keywords:** produce prescription, food security, hypertension, diabetes, rural health, food is medicine

## Abstract

**Introduction:**

In the United States, over one in every ten households experiences food insecurity. Food insecurity is associated with often co-occurring adverse health consequences, including risk for obesity, type 2 diabetes, and hypertension. Within the “Food is Medicine” intervention space, Produce Prescription Programs (PRx) seek to alleviate food insecurity and improve diet and health outcomes by leveraging access to produce through healthcare organizations. Though these programs are burgeoning across the United States, research surrounding their implementation and outreach is limited.

**Methods:**

This study evaluates the implementation, reach, engagement, and retention of a PRx program piloted in two regions of Georgia (US) from 2020 to 2022. The study included 170 people living with one or more cardiometabolic conditions recruited from clinical sites in metropolitan and rural areas. The program provided pre-packaged produce boxes and nutrition education over six months. We examine participants’ baseline demographics, food security status, dietary patterns, and loss to follow-up across contexts (metropolitan and rural). We employ regression analyses and model comparison approaches to identify the strongest predictors of loss to follow-up during the pilot period.

**Results:**

In the pilot period of this program, 170 participants enrolled across rural and metropolitan sites. Of these, 100 individuals (59%) remained engaged for the six-month program. While many individuals met the target criteria of living with or at-risk of food insecurity, not all lived with low or very low food security. Metropolitan participants, males, and those with children in the household had significantly higher odds of loss to follow-up compared to rural participants, females, and those without children in the household. No other significant demographic or household differences were observed.

**Discussion:**

This study demonstrates the potential of PRx programs to enhance food and nutrition security and cardiometabolic health in metropolitan and rural clinical settings. Future research should focus on addressing barriers to engagement and expanding the reach, impact, and sustainability of PRx programs across diverse contexts.

## 1 Introduction

In the United States, over one in every ten households experienced food insecurity in 2022 (1). Food insecurity generally refers to a phenomenon in which individuals lack “physical, social, or economic access to sufficient, safe, and nutritious food that meets their dietary needs and food preferences for an active and healthy lifestyle” (2). Food insecurity in the United States has generally declined over the past decade; however, estimates of food insecurity prevalence in 2022 suggest an upending of this trend (1). As population-based studies demonstrate, food insecurity disproportionately affects households with incomes below the federal poverty line and those who identify as Black or Hispanic (1). Food insecurity is often accompanied by a myriad of adverse outcomes, including unstable housing (3, 4), lack of transportation (5), and physical and mental health outcomes (6–8).

With mounting evidence of its adverse consequences for well-being, scholars and public health practitioners view food insecurity as a pressing healthcare issue in the 21st century, particularly for combatting diet-related cardiometabolic diseases (9). Cumulatively, annual diet-related cardiometabolic diseases cost an estimated $301 per person—$50.4 billion for the US population (10). Disparities are evident in diet quality and the burden of cardiometabolic diseases, which disproportionately affect marginalized and minoritized communities, including individuals who identify as Black or African American and those living with low socioeconomic status (11). These disparities are complex, multidimensional, and potentially synergistic in their effects (12). For instance, after adjusting for socioeconomic status and other known risk factors, non-Hispanic Black individuals experience an excess burden of cardiovascular diseases compared to other racialized groups (13). The experience of food insecurity is intertwined within this nexus and shows robust associations with both poor diet quality and adverse cardiometabolic outcomes (14).

In the US, the prevalence of food insecurity is statistically significantly higher in principal cities in metropolitan areas (urban) and nonmetropolitan areas (rural) than in metropolitan areas outside principal cities (1). Using data from the Current Population Survey Food Security Supplement (CPS-FSS), US-wide prevalence estimates are similar in principal cities (15.3%) and nonmetropolitan areas (14.7%) (1). In more granular analyses, Gundersen et al. previously documented the highest average rate of food insecurity in the South region in nonmetro areas with an urban population of 20,000 or more not adjacent to a metro area (15). Despite similar prevalence estimates, researchers have documented differences in lived experiences with food insecurity across urban and rural areas. For instance, Morton et al. found higher engagement with formal redistribution networks, like Supplemental Nutrition Assistance Program benefits or food banks and pantries, in urban food deserts. In contrast, more informal resource-sharing and reciprocity were more prevalent in rural food deserts across four counties in Iowa (16). More recent research by Byker-Shanks et al. revealed opposing findings, such that formal support systems were used more often than informal support systems among individuals in rural, low-income counties in six states (17). Gundersen et al. also report a greater number of food providers in remote rural counties than in large metropolitan counties (15). Additional barriers to food insecurity, such as poverty, access to transportation and availability, accessibility, and cost of nutritious foods, may all disproportionately impact rural areas (17–19). Variation in these factors may also impact health outcomes, including associations between household food insecurity and quality of life metrics among rural but not urban women (20). As such, it is important to examine how the area of implementation (metropolitan versus rural) impacts outreach, engagement, and retention in the context of nutrition interventions.

Based on the well-established relationships between food insecurity, diet quality, and cardiometabolic outcomes, healthcare systems across the US are increasingly adopting “Food is Medicine” interventions to improve well-being (21, 22). Food is Medicine (FIM) interventions generally refer to a range of programs and services that address the links between nutrition and health; these programs may provide food vouchers, fresh foods, or nutrition education and healthcare services through multisectoral partnerships (22). Within this suite of interventions, Produce Prescription (PRx) Programs have emerged as a healthcare-based approach to improve diet quality and health outcomes, particularly among individuals living with low household incomes or food insecurity (23). As framed by Mozaffarian et al., these interventions range from more intensive, treatment-based approaches (e.g., medically tailored meals) to broader, preventative approaches (e.g., population-level policies and programs such as the Supplemental Nutrition Assistance Program) (24). In this schema, medically tailored meal programs target patients with more complex chronic diseases and high healthcare utilization. In contrast, produce prescriptions (PRx) target a broader subset of individuals living with or at risk for diet-related conditions (24). Though many FIM programs began on a localized or regional level, the allocation of federal funding to support the expansion of these programs is increasing. As a result, research on PRx programs is comparatively more prolific than medically tailored meal or grocery program research (21).

Despite growing interest and investment in these programs, research on their implementation and reach remains limited in scope and study quality. Furthermore, few studies explore program engagement in rural areas or regions most affected by food insecurity and cardiometabolic disease—namely, the Southeast (25–27). PRx programs vary widely in implementation, structure, and evaluation. For instance, programs may include vouchers to farmers’ markets, grocery stores, or pre-packaged produce boxes, occasionally combined with nutrition or cooking education (22). A recent systematic review also highlights the heterogeneity in PRx duration, with peer-reviewed literature published on programs that ranged from several weeks to 18 months (25). Results from these studies suggest that PRx programs are often effective at improving food security and some components of dietary quality, with more limited evidence for other intended health outcomes. Our team’s previous research on Food is Medicine in a metropolitan safety-net hospital in Georgia observed a retention rate of 76.7% and significant decreases in food insecurity and diastolic blood pressure (26).

Moreover, relatively few articles have outlined the programmatic development and initial implementation of food prescription programs (27, 28). As these programs continue to grow across the US, so does the imperative to detail implementation and intended programmatic mechanisms of change. As Newman et al. note in their recent review of PRx programs, outlining key program characteristics and evaluation approaches may provide a valuable blueprint for new and existing programs (28). This study aims to sketch such a blueprint by describing and analyzing the initial development, implementation, and engagement of a Georgia-based PRx program that included participants from metropolitan and rural counties in two areas of the state. Specifically, we assess demographic characteristics, household composition, food security status, and baseline dietary intakes of enrolled participants. We then apply logistic regression analysis to identify predictors of loss to follow-up within metropolitan and rural cohorts. Our goal is to provide evidence-based insights that can guide the development and implementation of future programs, emphasizing inclusivity and equity.

## 2 Materials and methods

### 2.1 Local contexts and partnership roles

This study examines the implementation, engagement, and retention of a pilot PRx program at two sites in rural South Georgia and one in Metropolitan Atlanta between March 2021 and December 2022. In South Georgia, providers and healthcare workers affiliated with a regional healthcare system recruited participants. Most participants received a program referral through specialty clinics affiliated with the health system. However, referrals also occurred through their primary care clinics or word-of-mouth. Program staff informed healthcare providers at the partner clinics and health system that the program could serve any patient living with diabetes, coronary artery disease, or hypertension who may be low-income or living with food insecurity. At the time of the pilot program, the health partner in South Georgia had yet to implement social determinants of health screening, which would have otherwise informed the recruitment process. Most program participants in South Georgia resided in Tift or surrounding counties, including Berrien, Cook, Colquitt, and Lowndes. Participants from Metropolitan Atlanta were veterans recruited exclusively from Veterans Affairs programs, and most participants resided in nearby Fulton or Dekalb County. Open Hand Atlanta, the coordinating and administrating agency of this program, is a community-based organization located in Atlanta with satellite services across other regions of Georgia. Emory University served as the research and evaluation partner for this pilot program.

### 2.2 Intervention approach

Like many PRx programs, this program adopted a multi-component approach incorporating nutrition and cooking education, access to produce, and regular touchpoints with a registered dietitian and community health worker over 6 months. Specifically, the program included a six-week evidence-based Cooking Matters® nutrition education curriculum led by a registered dietitian and cooking instructor. For the initial 6 weeks of the program, participants met weekly for approximately 2 hours of nutrition and cooking education. In the first hour, the registered dietitian covered topics such as constructing healthy meals on a budget, reading nutrition labels, and following dietary recommendations. In the second hour of the class, participants practiced cooking skills by preparing a healthy meal that they were encouraged to consume with one another. In addition to these classes, participants received a produce box weekly. The content of produce boxes varied according to the season and location, but each box contained produce valued at approximately $25. Select participants in South Georgia also received a gas card (valued at $20 per week per household) or transportation voucher to alleviate transportation barriers reported in previous PRx intervention studies. The record of which participants received gas cards and their total value was not prioritized in this evaluation, limiting our ability to assess whether receipt impacted program participation or outcomes.

Participants who attended at least four of the six weeks of classes were eligible to continue receiving produce weekly for four additional months. In this study, we characterize participants as graduates if they remained engaged by picking up produce weekly from the healthcare service partner site and attending monthly reunion sessions over the intervention period. Monthly reunion sessions provided participants with a the space to interact and engage with the registered dietitian, cooking instructor, and, in some cases, a community health worker. At these monthly reunion sessions, the registered dietitian addressed additional topics, including food safety, micronutrients and “eating the rainbow,” and heart-healthy diets. Often, topics were selected based on participants’ interests to adopt a participant-led and tailored approach. In addition, cooking tools, such as electric skillets and vegetable choppers, were provided at graduation and reunion sessions to aid with self-sufficiency in the kitchen. During the study period, 14 cohorts enrolled in the program across South Georgia and Metropolitan Atlanta. The average cohort size at baseline across all cohorts was 13 individuals. Of note, cohorts were intentionally designed to be small 10–15 participants to ensure in-person activities could accommodate social distancing.

**Figure 1:**
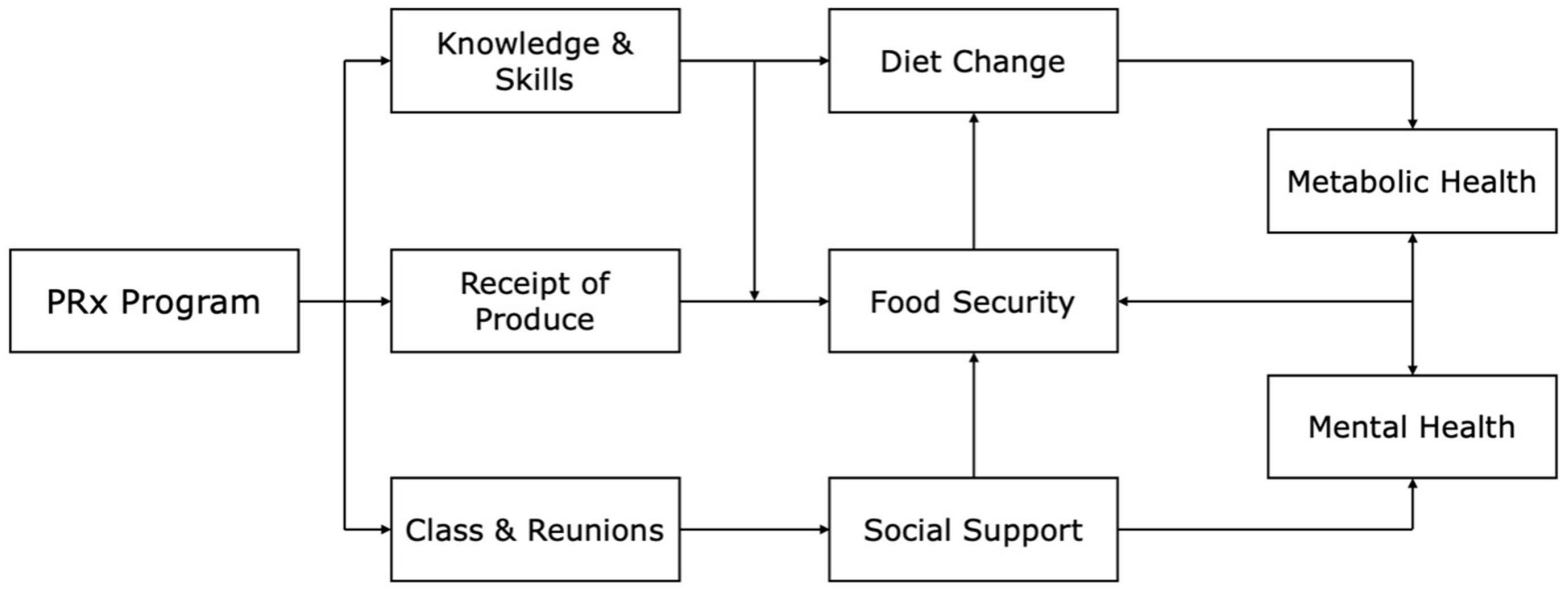
Produce prescription program inputs and theory of change.

The PRx program detailed in this study had five stated goals, which relate to the pathways of the theory of change shown in Figure 1: (1) improve food security; (2) increase fruit and vegetable consumption; (3) reduce consumption of fried foods and sugar-sweetened beverages; (4) increase confidence with procuring and preparing healthy foods; and (5) improve physical health.

### 2.3 Survey methods

**Figure 2:**
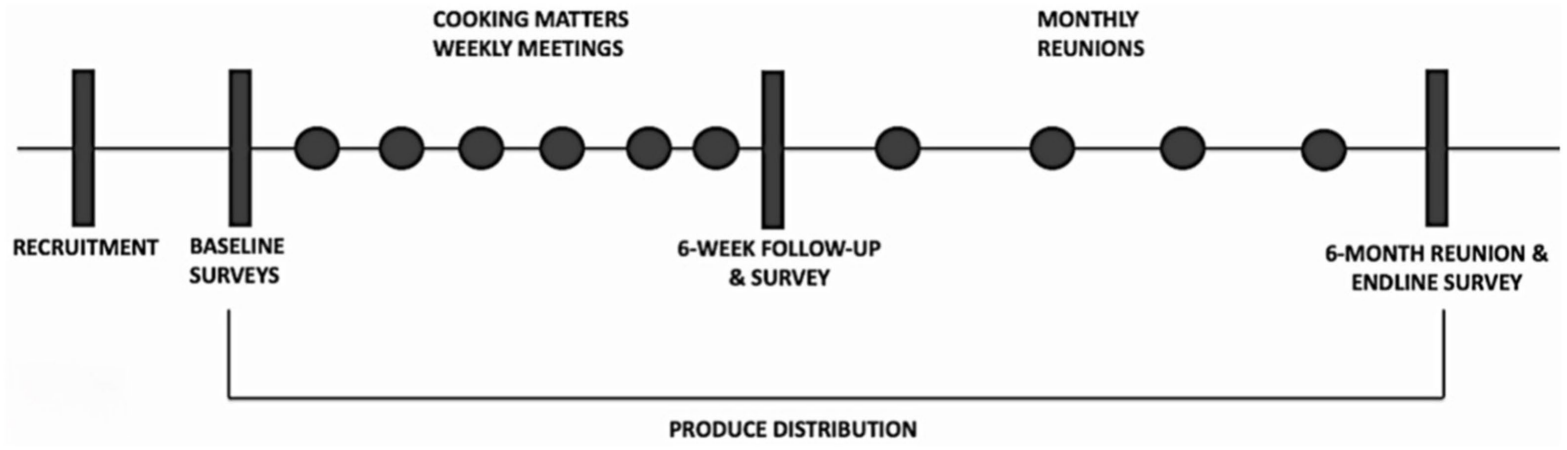
Survey timepoints to facilitate program monitoring and evaluation.

To assess program effectiveness, we conducted surveys the week before the first Cooking Matters® class (baseline measures), at the end of the last Cooking Matters® class (6 weeks/midline), and at the end of the last monthly reunion (6 months/endline), as outlined in Figure 2.

The surveys captured sociodemographic information, medication adherence and hospitalization, household food security, diet, food resource management and purchasing practices, and attitudes and confidence across different domains of dietary-related behaviors. Sociodemographic information collected included age in years, sex (male/female), self-identified race and ethnicity, household size, approximate monthly household income, and household member participation in food assistance programs (including Special Supplemental Nutrition Program for Women, Infants, and Children (WIC), SNAP, free or reduced-price school meals, or food pantries or food banks). Household-level food security was measured using the validated United States Department of Agriculture (USDA) 6-item household food security survey module (HFSSM) (29).

We used two self-report methods to assess dietary intakes: a nonquantitative 24-hour fruit and vegetable recall and an abbreviated food frequency questionnaire (FFQ) that queried consumption of the following: fruits, green salads, dark greens, non-fried vegetables, fried potatoes, white potatoes, and beans. Likert scale response options for the food frequency questionnaire included ‘Not at all,’ ‘Once a week or less,’ ‘More than once a week,’ ‘Once a day,’ and ‘More than once a day.’ We selected this FFQ because of its relatively low participant burden; it is also a validated and approved metric for programs receiving funding from SNAP-Ed. Data from this abbreviated FFQ were used to construct a dietary index score. We coded responses for foods assessed as healthier options (fruits, green salads, dark greens, non-fried vegetables, and beans) from 0 (Not at all) to 4 (More than once a day). Fried potatoes and white potatoes were reverse-coded. We then calculated a sum score across each item and scaled the sum to represent the proportion of the total attainable score (28 for the highest possible frequency of healthy food group consumption). This score was then multiplied by 10 to improve interpretability.

### 2.4 Statistical methods

We use descriptive statistics, including means, frequencies, and categorical tabulations, to assess baseline characteristics and loss to follow-up of participants in a pilot PRx program. We use Chi-square or Fisher’s exact tests to assess differences across unpaired categorical variables, such as the comparisons between participants who remained engaged in the program and those lost to follow-up and those in rural versus metropolitan areas. We apply logistic regression to model predictors of loss to follow-up and use model comparison approaches to identify the strongest predictors of loss to follow-up. In regressions, we collapse categorical variables with few observations. Specifically, we binarize employment as working or non-working and monthly household income as less than $1700 or greater than $1700. We selected a monthly household income threshold of $1700 as an approximation of the U.S. Department of Health and Human Services’ poverty guideline, which indicates annual incomes below $20,440 for households comprised of two individuals.

## 3 Results

The program enrolled 170 participants in the initial pilot period. As displayed in Table 1, most participants during the pilot period were part of the rural cohorts (68%). Overall, most participants identified as Black or African American (57%), were aged 50 and over (73%), and identified as female (64%). There were statistically significant demographic differences between metropolitan and rural contexts; including, differences in racial identity (Fisher’s Exact, *p* < 0.001), sex (*χ*^2^ = 8.97, *p* = 0.003), and collapsed categorical income (*χ*^2^ = 24.45, *p* < 0.001). Specifically, a higher proportion of individuals from the VA cohorts in metropolitan Atlanta identified as Black or African American, male, and reported a relatively higher income (monthly income greater than or equal to $1700) compared to individuals enrolled in the rural cohorts. Most participants were retired or receiving Social Security Disability Insurance (SSDI or Disability) and lived with monthly household incomes of $2,000 or less (63%), amounting to approximately $24,000 or less annually. Most participants (65%) resided in households in which at least one individual utilized one or more public assistance food programs, the most common of which was SNAP (52%), followed by support from a local food pantry (16%). Food assistance utilization was significantly higher among individuals enrolled in rural cohorts compared to those in metropolitan cohorts (*χ*^2^ = 9.72, *p* = 0.002).

**Table 1:** Baseline sociodemographic characteristics of participants by program completion status and cohort context.

	Completer		LTF^1^		Overall	
	Metro (*N* = 24)	Rural (*N* = 76)	Metro (*N* = 31)	Rural (*N* = 39)	Metro (*N* = 55)	Rural (*N* = 115)
Racial identity					Fisher’s Exact *p* < 0.001 ***	
American Indian	2 (8%)	2 (3%)	2 (6%)	1 (3%)	4 (7%)	3 (3%)
Asian or Asian American	1 (4%)	0 (0%)	1 (3%)	2 (5%)	2 (4%)	2 (2%)
Black or African American	16 (67%)	36 (47%)	27 (87%)	18 (46%)	43 (78%)	54 (47%)
Hawaiian, Pacific Islander	0 (0%)	0 (0%)	0 (0%)	1 (3%)	0 (0%)	1 (1%)
White or Caucasian	5 (21%)	38 (50%)	1 (3%)	16 (41%)	6 (11%)	54 (47%)
Missing	0 (0%)	0 (0%)	0 (0%)	1 (3%)	0 (0%)	1 (1%)
Age						
Under 18	0 (0%)	0 (0%)	0 (0%)	1 (3%)	0 (0%)	1 (1%)
18–29	1 (4%)	3 (4%)	2 (6%)	2 (5%)	3 (5%)	5 (4%)
30–39	3 (12%)	5 (7%)	3 (10%)	3 (8%)	6 (11%)	8 (7%)
40–49	4 (17%)	13 (17%)	4 (13%)	2 (5%)	8 (15%)	15 (13%)
50–59	6 (25%)	21 (28%)	5 (16%)	12 (31%)	11 (20%)	33 (29%)
60 and over	10 (42%)	34 (45%)	17 (55%)	19 (49%)	27 (49%)	53 (46%)
Sex					*χ*^2^ = 8.97, *p* = 0.003**	
Female	15 (62%)	57 (75%)	11 (35%)	26 (67%)	26 (47%)	83 (72%)
Male	9 (38%)	19 (25%)	20 (65%)	13 (33%)	29 (53%)	32 (28%)
Education						
Less than a high school degree	0 (0%)	17 (22%)	3 (10%)	13 (33%)	3 (5%)	30 (26%)
High school or ged certificate	2 (8%)	30 (39%)	7 (23%)	13 (33%)	9 (16%)	43 (37%)
Two-year college or technical school degree	10 (42%)	12 (16%)	10 (32%)	6 (15%)	20 (36%)	18 (16%)
Some college/technical school, but have not graduated	3 (12%)	14 (18%)	2 (6%)	4 (10%)	5 (9%)	18 (16%)
Four-year college or technical school degree	5 (21%)	2 (3%)	6 (19%)	2 (5%)	11 (20%)	4 (3%)
More than four-year college degree	3 (12%)	1 (1%)	3 (10%)	1 (3%)	6 (11%)	2 (2%)
Missing	1 (4.2%)	0 (0%)	0 (0%)	0 (0%)	1 (1.8%)	0 (0%)
Employment						
Not employed/Homemaker	3 (12%)	10 (13%)	3 (10%)	4 (10%)	6 (11%)	14 (12%)
On disability	9 (38%)	34 (45%)	9 (29%)	19 (49%)	18 (33%)	53 (46%)
Retired	9 (38%)	22 (29%)	10 (32%)	8 (21%)	19 (35%)	30 (26%)
Student	1 (4%)	0 (0%)	0 (0%)	2 (5%)	1 (2%)	2 (2%)
Working full-time	0 (0%)	3 (4%)	3 (10%)	2 (5%)	3 (5%)	5 (4%)
Working part-time	1 (4%)	5 (7%)	4 (13%)	3 (8%)	5 (9%)	8 (7%)
Other	1 (4%)	2 (3%)	2 (6%)	1 (3%)	3 (5%)	3 (3%)

*p < 0.05; **p < 0.01, ***p < 0.001. ^1^LTF indicates loss to follow-up.

Not all participants who started the program completed it (100/170). Demographic differences at baseline existed between those who completed the program and those who were lost to follow-up. As shown in Table 1, a greater proportion of individuals who identified as Black or African American were lost to follow-up, as were individuals who identified as male. Approximately half of all program graduates were non-Hispanic Black (52%), while the majority were female (72%) and aged 50 or older (71%). In addition, 41% of program graduates received Disability as their primary income, and 28% were retired.

The average household size reported was approximately three individuals, with about 20% of individuals residing in households with at least one child. The largest proportion of program graduates reported receiving public insurance (Medicare, Medicaid, etc.), whereas 16% reported having no health insurance. Approximately 25% of all program graduates reported monthly household incomes of less than $1,001, and most (59%) reported monthly household incomes of less than $1,700 (an approximate annual income of less than $20,400) (Table 2).

**Table 2:** Household characteristics of participants by program completion status and cohort context.

	Completer		LTF^1^		Overall	
	Metro (*N* = 24)	Rural (*N* = 76)	Metro (*N* = 31)	Rural (*N* = 39)	Metro (*N* = 55)	Rural (*N* = 115)
Monthly income					*χ*^2^ = 24.45, *p* < 0.001***	
Less than $1,001	4 (17%)	19 (25%)	3 (10%)	12 (31%)	7 (13%)	31 (27%)
$1,001–$2000	5 (21%)	35 (46%)	9 (29%)	20 (51%)	14 (25%)	55 (48%)
$2001–$3,000	4 (17%)	12 (16%)	3 (10%)	5 (13%)	7 (13%)	17 (15%)
More than $3,000	9 (38%)	3 (4%)	12 (39%)	0 (0%)	21 (38%)	3 (3%)
Do not Know	2 (8%)	3 (4%)	4 (13%)	1 (3%)	6 (11%)	4 (3%)
Missing	0 (0%)	4 (5.3%)	0 (0%)	1 (2.6%)	0 (0%)	5 (4.3%)
Receive food assistance					*χ*^2^ = 9.72, *p* = 0.002	
	12 (50%)	53 (70%)	14 (45%)	31 (79%)	26 (47%)	84 (73%)
Household size						
Mean (SD)	1.5 (± 0.74)	2.3 (± 1.5)	2.2 (± 1.8)	2.3 (± 1.1)	1.9 (± 1.5)	2.3 (± 1.4)
Missing	2 (8.3%)	4 (5.3%)	2 (6.5%)	1 (2.6%)	4 (7.3%)	5 (4.3%)
Any children	5 (21%)	18 (24%)	7 (23%)	14 (36%)	12 (22%)	32 (28%)
Missing	1 (4.2%)	2 (2.6%)	1 (3.2%)	5 (12.8%)	2 (3.6%)	7 (6.1%)
Any elderly	6 (25%)	26 (34%)	11 (35%)	11 (28%)	17 (31%)	37 (32%)
Missing	1 (4.2%)	2 (2.6%)	3 (9.7%)	5 (12.8%)	4 (7.3%)	7 (6.1%)

*p < 0.05; **p < 0.01, ***p < 0.001. ^1^LTF indicates loss to follow-up.

At baseline, 40% of participants were living with food insecurity—a rate almost four times higher than the national average. More granularly, 24% of individuals enrolled were living with low food security and 17% with very low food security. There were no statistically significant differences in baseline food security status between program graduates and those who were lost to follow-up, nor were there statistically significant differences in food insecurity between rural and metropolitan program sites (Table 3).

**Table 3:** Baseline food security and food assistance characteristics by program completion status and cohort context.

	Completer		LTF^1^		Overall	
	Metro (*N* = 24)	Rural (*N* = 76)	Metro (*N* = 31)	Rural (*N* = 39)	Metro (*N* = 55)	Rural (*N* = 115)
Food Insecurity Score						
Mean (SD)	2.13 (2.07)	1.78 (2.11)	2.00 (1.82)	1.59 (1.85)	2.06 (1.92)	1.71 (2.02)
Missing	1 (4.2%)	0 (0%)	1 (3.2%)	0 (0%)	2 (3.6%)	0 (0%)
3-level classification						
High or marginal food security	13 (54%)	45 (59%)	17 (55%)	23 (59%)	30 (55%)	68 (59%)
Low food security	5 (21%)	17 (22%)	8 (26%)	11 (28%)	13 (24%)	28 (24%)
Very low food security	5 (21%)	14 (18%)	5 (16%)	5 (13%)	10 (18%)	19 (17%)
Missing	1 (4.2%)	0 (0%)	1 (3.2%)	0 (0%)	2 (3.6%)	0 (0%)
Binary classification						
Food insecure	10 (42%)	31 (41%)	13 (42%)	16 (41%)	23 (42%)	47 (41%)
Not food insecure	13 (54%)	45 (59%)	17 (55%)	23 (59%)	30 (55%)	68 (59%)
Missing	1 (4.2%)	0 (0%)	1 (3.2%)	0 (0%)	2 (3.6%)	0 (0%)

*p < 0.05; **p < 0.01, ***p < 0.001. ^1^LTF indicates loss to follow-up.

At baseline, most individuals reported consuming salads, greens, non-fried vegetables, fried foods, potatoes, beans, meals away from home, and juices, on average, once a week or less. The few exceptions to this reporting pattern include consumption of fruits, sugar-sweetened beverages, and water, which were, on average, consumed with more frequency. Using these measures, overall dietary quality scores (ranging from 0–10) were equivalent for program graduates and those who were lost to follow-up (Table 4). Using the dietary recall activity, the average number of vegetables consumed in the previous 24 hours at baseline was also comparable among program graduates and those lost to follow-up (1.93 ± 1.69 and 1.96 ± 1.70, respectively), as were number of fruits (1.48 ± 1.43 and 1.68 ± 1.71, respectively). While the average number of unique fruits and vegetables consumed in the previous 24 hours at baseline were both higher in the metropolitan group, the difference across contexts was not statistically significant.

**Table 4:** Baseline dietary behaviors by program completion status and cohort context.

	Completer		LTF^1^		Overall	
	Metro (*N* = 24)	Rural (*N* = 76)	Metro (*N* = 31)	Rural (*N* = 39)	Metro (*N* = 55)	Rural (*N* = 115)
Unique fruits						
Mean (SD)	1.83 (1.49)	1.37 (1.40)	1.81 (1.76)	1.57 (1.68)	1.82 (1.63)	1.44 (1.49)
Median [Min, Max]	1.50 [0, 5.00]	1.00 [0, 7.00]	2.00 [0, 6.00]	1.00 [0, 8.00]	2.00 [0, 6.00]	1.00 [0, 8.00]
Missing	0 (0%)	1 (1.3%)	0 (0%)	2 (5.1%)	0 (0%)	3 (2.6%)
Unique vegetables						
Mean (SD)	2.04 (1.76)	1.89 (1.68)	2.35 (1.89)	1.62 (1.46)	2.22 (1.82)	1.80 (1.61)
Median [Min, Max]	2.00 [0, 5.00]	2.00 [0, 7.00]	2.00 [0, 6.00]	1.00 [0, 6.00]	2.00 [0, 6.00]	2.00 [0, 7.00]
Missing	0 (0%)	2 (2.6%)	0 (0%)	2 (5.1%)	0 (0%)	4 (3.5%)
Overall diet score						
Mean (SD)	4.72 (1.21)	4.83 (1.39)	4.86 (1.19)	4.74 (1.40)	4.80 (1.19)	4.80 (1.39)
Median [Min, Max]	4.46 [2.86, 7.50]	4.64 [1.43, 8.57]	5.00 [2.86, 7.14]	4.64 [0, 7.50]	4.64 [2.86, 7.50]	4.64 [0, 8.57]

*p < 0.05; **p < 0.01, ***p < 0.001. ^1^LTF indicates loss to follow-up.

To investigate predictors of loss to follow-up, we first created a multivariable logistic regression model that included all hypothesized sociodemographic predictors. In this model, as shown in Table 5, there were no statistically significant demographic or household composition differences observed between program graduates and those who were lost to follow-up; we display the model only for participants with full data (*n* = 146) to facilitate model comparison. Overall, the sociodemographic characteristics included in the logistic regression explained a marginal degree of the variability in loss to follow-up. We then applied a backward stepwise variable selection approach on complete data to determine the subset of variables that produce the best performing model. We used the Akaike information criterion (AIC), an estimation of model prediction error, to guide model selection. The variables retained as the best predictors of loss to follow-up were cohort context, sex, and whether there were children in the household. All other variables fell out of the final model.

**Table 5:** Odds of attrition by sociodemographic and household-level predictors.

Predictors	Loss to follow-up		
	Odds ratios	CI	*p*
Context [Rural]	0.42	0.16–1.03	0.059
Sex [Male]	1.78	0.83–3.85	0.141
Racial Identity [White]	0.62	0.26–1.40	0.250
Age [Under 50]	1.06	0.45–2.55	0.900
Employment [Working]	2.20	0.68–7.51	0.191
Monthly income [Greater than $1701]	0.71	0.29–1.70	0.446
Monthly income [Do not know]	1.45	0.31–6.95	0.634
FIS score^1^	0.95	0.78–1.15	0.609
Household size	0.96	0.60–1.44	0.837
Any children in household	1.38	0.78–2.62	0.286
Any adults over 65 in household	1.06	0.59–1.87	0.844
Observations	146		
R^2^ Tjur	0.118		

*p* < 0.05; **p < 0.01, ***p < 0.001. ^1^FIS score indicates food insecurity score from the USDA 6-item scale.

In this best-fit model, shown in Table 6, being part of a rural cohort predicted significantly lower odds of loss to follow-up [aOR: 0.37, CI: 0.18–0.79, *p* = 0.01]. Identifying as male predicted higher odds of loss to follow-up [aOR: 1.78, CI: 0.85–3.73], as did having children in the household [aOR: 1.41, CI: 0.97–2.10].

**Table 6:** Odds of attrition by sociodemographic and household-level predictors, retaining only predictors of best-fit.

Predictors	Loss to follow-up		
	Odds ratios	CI	*p*
Context [Rural]	0.37	0.18–0.79	0.010**
Sex [Male]	1.78	0.85–3.73	0.128
Children	1.41	0.97–2.10	0.079
Observations	146		
R^2^ Tjur	0.089		

*p < 0.05; **p < 0.01, ***p < 0.001.

## 4 Discussion

Of the 170 individuals enrolled in a PRx program between 2021 and 2022, 59% remained engaged for the six-month program period. Further data on loss to follow-up is needed to elucidate unobserved barriers to participation or areas for programmatic improvement. Integrating nutrition and health is an emerging priority in federal legislation, including the 2022 White House Strategy on Hunger and Nutrition. Over the past decade, various Food is Medicine program models have emerged to improve food security, nutrition, and health by leveraging existing healthcare infrastructure. However, studies of program implementation and evidence of implementation, enrollment, and retention across metropolitan and rural contexts remains relatively sparse. Furthermore, few existing studies assess program engagement and effectiveness in rural, underserved populations. Those living in rural areas of the Southeast may face more transportation barriers and different food environments than those in more urban regions, including metropolitan Atlanta. Yet, in this pilot program, retention was significantly higher among those in rural areas. Given limitations in the data collected, parsing the degree to which this difference is explained by underlying sample differences (Veterans in the metropolitan area) is not feasible. However, when controlling for related variables including sex, racial identity, and socioeconomic status, rurality remains the strongest predictor of retention. Analyses of how local contexts come to shape experience with food and food interventions will be critical for expanding PRx programs into more diverse communities. The initial stages of the pilot program served the targeted population, namely individuals living with food insecurity and diet-related cardiometabolic conditions; however, the sizable proportion of individuals living with high or marginal food security suggests that more refined screening processes for food insecurity may function to meet those most in need.

Based on a recent brief review by Newman et al., the PRx program reported on in this study aligns with many core elements of other programs, including health-based criteria for enrollment and the creation of an interface with a healthcare provider (28). While some programs require that participants meet with a health provider in a separate clinical encounter, a community health worker supported this PRx program and was available at each session. In effect, this created a “one-stop shop” for participants to gain nutrition education, access food, and ask health-related questions to a health worker who could directly connect participants in need with a clinical-based healthcare provider. Similarly, this program adopted the model of providing pre-packaged boxes of produce, which may be easier for programs operating outside city limits and with limited access to supermarkets or farmers’ markets. However, pre-packaged meals come with a trade-off, offering less potential autonomy or dignity regarding food choices and preferences among participants in comparison with a voucher-based model. Finally, among programs that provided data on retention, Newman and colleagues report a range from 62–100%. Pooling data from rural and metropolitan program contexts, we found a comparatively lower retention rate of 59%. The retention rate in this study is also lower than that documented in previous PRx programs administered, in part, by our study team in metropolitan Atlanta, which informed the design and implementation of this pilot program (26). The documented lower-than-average reported retention rate may be due to reporting bias or a temporal effect of the broader social and economic context of food and well-being during the pilot period of this program.

Notably, the pilot years of this program (2021–2022) coincided with the ongoing COVID-19 pandemic, food price inflation, and other externalities that may have undermined engagement and enrollment, particularly given that the target population of the intervention also faced substantive risk from infection. Nevertheless, this PRx program enrolled 170 participants during this period, with an average cohort size of 13 individuals. The significantly greater loss to follow-up among males compared to females warrants further investigation. Analyses of trends in home cooking demonstrate that a greater proportion of self-identified females report cooking at home (30). Furthermore, while the percentage of males who report cooking at home has increased in recent years, changes vary by educational attainment. Specifically, Taillie reports that the percentage of males with less than a high school education who cook has remained stagnant over the past decade (30). More recent examinations of National Health and Nutrition Examination Survey data demonstrate that females continue to perform significantly more food procurement and preparation responsibilities in households in the United States (31). Gender norms surrounding cooking and feeding responsibilities may explain the greater loss to follow-up among men. Qualitative research on similar programs suggests that economic and structural barriers, such as limited income and medical concerns associated with disease management, may hinder program engagement (32). Though baseline health status based on BMI and blood pressure did not predict loss to follow-up, other unmeasured aspects of health, including mental well-being, other co-occurring conditions, or functional mobility limitations, may similarly affect program engagement. Additionally, though not assessed in this study, competing demands such as work or caregiving responsibilities may limit the ability to consistently attend program classes or pick up produce throughout the six month intervention period. This hypothesis is supported by the strong predictive power of loss to follow-up among households with children in this study. As such, building opportunities for childcare or parental-child PRx may further enable program participation among caregivers.

Healthcare and nonprofit organizations seeking to develop and implement a food or produce prescription program may benefit by first assessing provider and patient perceptions about the program, as demonstrated in several recent studies (33). Qualitative research during the early phases of program implementation may enable earlier identification of barriers to recruitment, enrollment, and participation. Additionally, implementing agencies should craft a plan for examining the effects of interventions and disseminating results widely through scholarly and community-based forums. Given that agencies may not have research capacities or funding for research, partnerships with academic institutions and scholars should be explored. Echoing Newman et al. it is vital for programs to publish or report on program processes, inputs, and outcomes to foster transparency in the intervention setting and enable comparisons across place. Enhancing scholarly ability to compare programs will ultimately aid in crafting informed, evidence-based strategies for future PRx programs in different contexts. Finally, since most of these programs operate within a finite duration, long-term program and outcome sustainability assessment should be prioritized in future research and program implementation.

## 5 Conclusion

This study demonstrates that produce prescription programs can successfully engage patients living with or at risk of food insecurity and cardiometabolic diseases in both rural and metropolitan areas. Though documented retention was lower than previously reported, the ability to coordinate, maintain, and retain engagement during the broader context of the global COVID-19 pandemic and associated macrosocial changes shows promise for the potential of future PRx programs. Further research on food and produce program effectiveness, dose, duration, and outcomes is sorely needed as these programs expand across the United States. Finally, these programs must not occur in isolation but should instead be interwoven with broader policy changes that provide people with resources to enable and support their well-being.

## Data availability statement

The datasets presented in this article are not readily available because the data are not publicly available due to the inclusion of patient health information. Requests to access the datasets should be directed to caroline.owens@tufts.edu.

## Ethics statement

The studies involving humans were approved by Emory Institutional Review Board. The studies were conducted in accordance with the local legislation and institutional requirements. Written informed consent for participation was not required from the participants or the participants’ legal guardians/next of kin because This project was deemed a program evaluation project for an existing and ongoing intervention and was deemed exempt from review.

## Author contributions

CO: Conceptualization, Investigation, Methodology, Project administration, Software, Supervision, Visualization, Writing – original draft, Writing – review & editing, Data curation, Formal analysis, Resources. MC: Conceptualization, Investigation, Methodology, Project administration, Supervision, Writing – review & editing. TR: Funding acquisition, Project administration, Resources, Supervision, Writing – review & editing. AM: Funding acquisition, Project administration, Resources, Supervision, Writing – review & editing. AW: Methodology, Supervision, Writing – review & editing.
